# Coadministration of kla peptide with HPRP-A1 to enhance anticancer activity

**DOI:** 10.1371/journal.pone.0223738

**Published:** 2019-11-08

**Authors:** Wenjing Hao, Cuihua Hu, Yibing Huang, Yuxin Chen

**Affiliations:** 1 Key Laboratory for Molecular Enzymology and Engineering of the Ministry of Education, Jilin University, Changchun, China; 2 School of Life Sciences, Jilin University, Changchun, China; 3 Ministry of Education Key Laboratory for Cross-Scale Micro and Nano Manufacturing, Changchun University of Science and Technology, Changchun, China; 4 International Research Centre for Nano Handling and Manufacturing of China, Changchun University of Science and Technology, Changchun, China; 5 JiangsuProteLight Pharmaceutical & Biotechnology Co., Ltd., Jiangyin, China; University of PECS Medical School, HUNGARY

## Abstract

The apoptosis-inducing peptide kla (KLAKLAK)_2_ possesses the ability to disrupt mitochondrial membranes and induce cancer cell apoptosis, but this peptide has a poor eukaryotic cell-penetrating potential. Thus, it requires the assistance of other peptides for effective translocation at micromolar concentrations. In this study, breast and lung cancer cells were treated by kla peptide co-administrated with membrane-active anticancer peptide HPRP-A1. HPRP-A1 assisted kla to enter cancer cells and localized on mitochondrial membranes to result in cytochrome C releasing and mitochondrial depolarization which ultimately induced apoptosis.The apoptosis rate was up to 65%and 45% on MCF-7 and A549 cell lines, respectively, induced by HPRP-A1 coadministration with kla group. The breast cancer model was constructed in mice, and the anticancer peptides were injected to observe the changes in cancer volume, andimmunohistochemical analysis was performed on the tissues and organs after the drug was administered. Both the weight and volume of tumor tissue were remarkable lower in HPRP-A1 with kla group compared with thosepeptidealonggroups. The results showed that the combined drug group effectively inhibited the growth of cancer and did not cause toxic damage to normal tissues, as well as exhibited significantly improvement on peptide anticancer activity *in vitro* and *in vivo*.

## Introduction

The global cancer burden has increased and statistics indicate that lung and breast cancers are the leading causes of mortality in men and women, respectively. At present, the treatment methods for cancer include surgery, radiotherapy, chemotherapy, and traditional chemical medicine. However, chemical drugs can damage normal tissues while killing cancer cells, and the cancer cells easily metastasize and cause relapse after treatment. Therefore, circumventing the drug resistance and non-specific toxicity of traditional drugs is a major challenge in cancer treatment[1–3]. Thus, there is an urgent need to develop new anticancer drugs to overcome the shortcomings of traditional drugs.

Compared with small molecule anticancer drugs, anticancer peptides can effectively overcome the immune barrier of the body in the course of drug treatment of cancer and better exert their effects[4]. Peptides are less toxic to normal cells and tissues and thus have a certain selectivity and tissue permeability[5–7]. Furthermore, peptide drugs can interact with functional proteins in the signalling pathways of cancer cells to inhibit their growth or cause apoptosis, killing cancer cells efficiently[3]. Hence, there is the potential to develop new anti-tumour peptide drugs with high activity, low toxicity, and high tissue permeability[5, 6, 8]. Apoptosis resistance and unlimited proliferation are among the main characteristics of cancer cells[9]. Therefore, inducing apoptosis of cancer cells is one of the major goals to treat cancer.

Mitochondria are the regulatory centres of apoptosis and cell death. In the study of anti-tumour drugs, mitochondria have become one of the targets of drug actions to induce apoptosis of cancer cells. Since the lipid composition of mitochondrial membranes closely resembles that of Gram-negative bacteria, various peptides with an antimicrobial activity against Gram-negative bacteria may also cause depolarization and/or disruption of mitochondrial membranes and act as apoptosis-inducing agents[10]. However, the uptake rate of these polypeptides by cancer cells is low due to the difference in the composition of the cell membrane and bacterial wall, contributing to their poor cell-penetrating potentials. As a result, these peptides cannot effectively bind to the mitochondria of cancer cells and exert their effects. Although some cationic polypeptides can induce apoptosis at high concentrations, the excessive concentration can cause greater toxicity to normal tissues. Hence, it is necessary to choose an effective method to deliver peptides with a mitochondrial targeting potential to cancer cells.

The most actively investigated mitochondrion-disrupting peptide, the kla peptide, has an α-helical structure when located in the membrane. It destroys the mitochondrial membrane and causes apoptosis, but this polypeptide has poor cell penetration and low utilization. Therefore, a strategy is needed to facilitate its penetrating ability to the cell and localization to mitochondria, so that kla can destroy the mitochondrial membrane and cause apoptosis even at a low concentration[11, 12]. After the discovery of the proapoptotic ability of kla, many efforts have been made to improve the therapeutic efficacy of this protein by improving its cell-penetrating ability. The most straightforward approach is to use an auxiliary peptide to facilitate kla entering cells[13].

Anticancer peptides (ACPs) have attracted widespread attention as a new class of anticancer drug candidates due to their high activity and unique mechanism of targeting cells. HPRP-A1 peptide, derived from the N-terminus of ribosomal protein L1 of *Helicobacter pylori*, has shown excellent antimicrobial activity and anticancer activity[14]. HPRP-A1 is an amphipathic α-helical peptide with potent antimicrobial activity against various Gram-positive and Gram-negative bacteria and negligible hemolytic activity against human red blood cells[15]. This peptide has been demonstrated to possess significant anticancer activity against many cancer cell lines. The α-helical membrane-active peptide disrupts the target cell membrane by a necrotic mechanism at high concentrations. In contrast, at low concentrations, it induces target cell apoptosis[16]. In addition, HPRP-A1 can assist other chemical drugs in passing the cytoplasmic membrane to concentrate within their intracellular targets[15].

In this study, we hypothesized that cell membrane disruption by α-helical anticancer peptides should increase the intracellular concentration of the kla peptide simultaneously, thus enhancing its anticancer effect. HPRP-A1 was employed together with kla to investigate the combined efficacy *in vitro* and *in vivo* and to delineate the mechanism of the synergistic action. The objectives of this study were three-fold: first, to explore the combined anticancer activity of peptides and chemotherapeutic drugs; second, to understand the synergistic mechanism of these two types of anticancer agents; and third, to verify the clinical potential of this new high efficacy, low toxicity approach to cancer chemotherapy.

## Materials and methods

A FITC Annexin V/PI apoptosis detection kit was purchased from BD Biosciences (NY, USA). Hoechst 33258 and PI dye detection kits were provided by Solarbio Life Science (Beijing, China). MitoTracker® probes and a Pierce^™^ BCA protein assay kit were provided by Thermo Fisher Scientific (MA, USA). FITC-klawas provided by GL Biochem Ltd. (Shanghai, China). MitoCapture Mitochondrial Apoptosis Detection Fluorometric Kit was provided by BioVision(SF,USA). The human breast cancer cell line MCF-7, lung cancer cell line A549, and mouse embryonic fibroblast cell line NIH3T3 were obtained from the Institute of Biochemistry and Cell Biology, Shanghai Institute for Biological Sciences, Chinese Academy of Sciences (Shanghai, China).

### Peptide synthesis and purification

The anticancer peptide HPRP-A1 (Ac-FKKLKKLFSKLWNWK-amide) and apoptosis inducing peptide kla (Ac-KLAKLAKKLAKLAK-amide) were synthesized using solid phase methods on MBHA rink amide resin by 9-fluorenyl-methoxycarbonyl chemistry as described previously[17] and the crude peptides were purified by preparative reversed-phase high-performance liquid chromatography (RP-HPLC) using a ZORBAX SB-C8 Stable Bond Analytical (4.6×150 mm) from Agilent Technologies (DE, USA) with a linear AB gradient (0.1% acetonitrile/min) at a flow rate of 1 mL/min, where eluent A was 0.1% aqueous trifluoroacetic acid (TFA) and eluent B was 0.1% TFA in acetonitrile and then,using the same RP-HPLC, a Zorbax 300 SB-C 8 column and eluent with a linear AB gradient (1% acetonitrile/min) at a flow rate of 1mL/min to analyse the pure peptide[18]. The molecular weight was determined by AB SCIEX TOF/TOF 5800 system.

### Circular dichroism spectroscopy of peptides

The helical structures of peptides were detected by circular dichroism (CD)spectroscopy using a J-810 spectropolarimeter (Jasco, Tokyo, Japan) at 25°C in KP buffer (50 mM KH_2_PO_4_/K_2_ HPO_4_ and 100 mM KCl, pH 7) and an α-helix-inducing solvent, TFE buffer (50 mM KH_2_ PO_4_/K_2_ HPO_4_ and 100 mM KCl, pH 7, in 50% TFE). The values of molar ellipticities of the peptides HPRP-A1, kla with or without HPRP-A1 at wavelengths of 208 and 222 nm were used to estimate the relative helicity of the peptides.

### Haemolysis assessment

The haemolysis assessment of peptides HPRP-A1, kla with or without HPRP-A1 was carried out as described previously[1, 15]. Briefly, 4 μM kla with or without HPRP-A1 at various concentrations were incubated at 37°C with rotation at 90rpm for 2 h in the presence of human red blood cells (RBCs) in PBS. PBS and distilled water were employed as negative and positive haemolysis controls, respectively.The plates were then centrifuged at 3000 rpm at 4°C for 10 min, and 90 μL of supernatant was transferred to a flat-bottomed 96-well plate. The supernatant absorbance at 540 nm was recorded. The haemolytic activity was defined as hemolysis (%) = (absorbance sample)—(absorbance blank) / (highest absorbance positive control (water)- absorbance blank) ×100.

### Cell culture

MCF-7, A549, and NIH3T3 cell lines were cultured in Dulbecco’s modified Eagle’s medium (DMEM) (Gibco Life Technologies, Thermo Fisher Scientific, MA, USA) containing fetal bovine serum (FBS) (10% v/v), penicillin (100 U/ml), and streptomycin (100 U/ml) at 37°C in a humidified atmosphere with 5% CO_2_.

### *In vitro* cytotoxicity evaluation

Cytotoxicity was assessed using MTT method. MCF-7, A549, and NIH3T3 cells were cultured in a 96-well culture plate for 24 h. Then, the cells were incubated with free HPRP-A1, kla with or without HPRP-A1 at various concentrations for 12 h at 37°C. MTT was then added (5 mg/ml, 20 μl per well), and cells were incubated for another 4 h. After removal of the supernatant, DMSO was added (150 μl per well) to dissolve the formazan crystals, and the absorption at492nm was measured using a microplate reader (GF-M3000; GaomiCaihong Analytical Instruments Co., Ltd., Shandong, China). The cell viability was calculated using the software Origin 6.0. The MTT assays were performed in triplicate.

### Cellular uptake and co-localization of the peptides

MCF-7 cells were cultured in glass-bottomed dishes for 24 h. For peptide cellular uptake studies, after 24 h of culture with DMEM containing 10% blood serum, cells were washed with PBS for 3 times and labelled with Hoechst 33258 for 30 min at 37°C. Then, cells were washed three times with PBS, and different concentrations of FITC-labelled kla with or without 4 μM HPRP-A1 were added to the dish for 1 h. Cells were then scanned by LSCM (LSM710, CarlZeiss, Oberkochen, Germany). The LSCM images were processed and analyzed using the ZEN lite 2012 image software, CarlZeiss, Oberkochen, Germany.

For the co-localization tests, the cells were stained with 200 nM MitoTracker ® Red FM to label mitochondria and Hoechst 33258 to label nuclei according to the manufacturer’s instructions and then treated with different concentrations of FITC-labelled kla with or without 4 μM HPRP-A1 and incubated for another 1 h. The stained cells were imaged using a LSCM. Composite images were created by overlapping the images obtained from individual channels.

### Apoptosis analysis by flow cytometry

For the apoptosis assay, an annexin V-FITC/PI double staining method was used. MCF-7 and A549 cells were treated with HPRP-A1 and kla with or without HPRP-A1 for 12 h. At the end of the treatment, the cells were trypsinized, washed with PBS and centrifuged at 1500rpmfor 5 min. Then, the cells were resuspended in 500 mL of binding buffer and stained with 5 μL annexin V-FITC and 5 μL PI. The cells were incubated in the dark at room temperature for 15 min. Finally, the stained cells were collected for flow cytometric analysis (Cytomics TM FC 500, Beckman Coulter, Miami, FL, USA).

### Mitochondrial depolarization

Apoptosis of MCF-7 cells was detected using a MitoCapture Mitochondrial Apoptosis Detection Fluorometric Kit. In healthy cells, MitoCaptureReagent accumulated and aggregated in the mitochondria, emitting a bright red fluorescence. In apoptotic cells, the reagent cannot accumulate in the mitochondria due to the altered mitochondrial membrane potential, and it remains in its monomericformin the cytoplasm and emits green fluorescence. MCF-7 cells were incubated with peptides HPRP-A1, kla and kla with or without HPRP-A1 6 hours, then, incubated with the MitoCapture reagent at 37°C for 15 minutesand observed using a fluorescence microscope (IX71).

### Cytochrome C releasing test

MCF-7 cells treated with kla with or without HPRP-A1 were washed with ice-cold PBS and digested in a trypsin-EDTA solution. Cells were collected by repeated centrifugation with ice-cold PBS. After centrifugation and incubating with Mitochondrial separation reagent on ice for 10–15 min, the cell suspension was oscillated through turbo oscillators and centrifuged at 30000rpm. The supernatant was cytoplasmic proteins, and precipitates were mitochondria. Precipitates were solubilized in RIPA lysis buffer for 1 h at 4°C. After centrifugation, the supernatant was collected, and protein concentrations were determined using a bicinchoninic acid (BCA) protein assay kit. Protein samples (120 μg) were separated on 15% SDS-PAGE gels and then transferred to polyvinylidene fluoride membranes. Membranes were blocked for 1 h with 5% dry skim milk in PBS at room temperature and blotted with the anti-Cytochrome C primary antibody overnight at 4°C. Anti-COX-IV and anti-tubulin primary antibodies were applied at a 1:5,000 dilution as a loading control. After washing with PBS containing Tween-20, the membranes were incubated with horse radish peroxidase-conjugated secondary antibodies for 1 h at room temperature. Blots were developed using an enhanced chemiluminescence kit and visualized by the Tanon-5200 Chemiluminescent Imaging System.

### *In vivo* antitumor efficacy evaluation

Six-8-week-old female BALB/c nude mice weighting 18–22 g purchased from Beijing Weitonglihua Co., Ltd. (Beijing, China) were housed in a good laboratory practice laboratory. MCF-7 cells (1 × 10^7^) were subcutaneously injected into the right posterior limb of mice. When the tumours grew to 150 mm^3^, the xenografted mice were randomly divided into four groups (n = 6 per group).

Based on in vitro results and a previous study, 10 mg/kg kla with or without 10 mg/kg HPRP-A1 were coadministrated directly into MCF-7 tumours in the xenografted mouse model. Two peptides were mixed well before injection. Tumours were directly injected with saline (control), 10 mg/kg kla alone, or 10 mg/kg kla with 10 mg/kg HPRP-A1 every 2 days for 16 days. The tumour size was calculated using the following formula:tumor size = (length × width^2^)/2. The inhibition rate of tumour growth (%) was calculated using the following formula: (tumour volume in control group − tumour volume in test group)/tumour volume in control group × 100%. At the end of the experiment, mice were sacrificed.

Tumour tissues and major organs (heart, liver, spleen, lungs, and kidneys) were dissected out for histological observation. The organs were fixed in 10% paraformaldehyde, embedded in paraffin, sectioned at a thickness of 5 μm, then stained with hematoxylin and eosin (H&E), and tumors were stained with a Ki67 antibody(Wanleibio, Shenyang, China) and then visualized with dye-conjugated secondary antibodies under an optical microscope. For transferase-mediated dUTP nick end-labelling (TUNEL) staining, detecting solution was used in the One Step TUNEL Apoptosis Assay Kit (Beyotime Bio, Shanghai, China)[19, 20]. All animal care and handling were performed in accordance with the guidelines of the Animal Ethics Committee of Jilin University (Approval No. JLUSWLL003; Jilin, China).

## Statistical analysis

Average data is presented as the mean ± SD of at least three independent experiments. Statistical significance of differences between groups were analyzed by t-test, with significance accepted at *P*< 0.05 (*), *P*< 0.01 (**) and *P*< 0.001 (***).

## Results

### Purification and basic physicochemical property of peptides

HPRP-A1 and kla peptides were purified by RP-HPLC and further confirmed by AB SCIEX TOF/TOF 5800 system as shown in (S1 Fig). Structural changes of the peptides after mixing were detected by CD. The CD spectra showed that the structuresof peptide mixture were significantly different from those of HPRP-A1 and kla alone as shown in (S2 Fig). In both hydrophilic and hydrophobic environments, the degree of alpha helix of polypeptide HPRP-A1 decreased when mixed with polypeptide kla. In addition, the haemolytic toxicity of peptides was in a safe range as shown in (S1 Table), the hemolytic concentration of polypeptide co-administration was much higher than that of active action.

### *In vitro* anticancer activity

**Fig 1:**
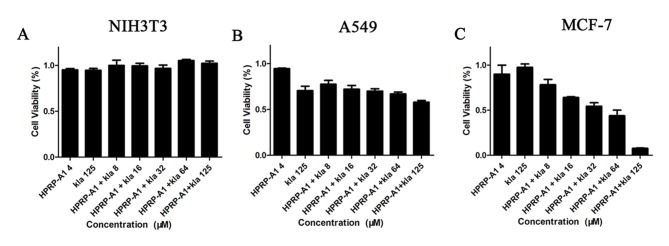
Cytotoxic effects of kla with or without HPRP-A1 against NIH3T3, A549, and MCF-7 cell lines. (A) Normal mouse cell line NIH 3T3, (B) human lung cancer line A549, and (C) human breast cancer cell line MCF-7 were treated with various concentrations of kla with or without 4 μM HPRP-A1 for 12 h, and then cell viability was detected by MTT assays.

To determine the anticancer activity of the peptides, the cell viability of A549, MCF-7 and NIH3T3 cell lineswas used detected using MTT method. Cells were treated with various concentrations of kla with or without 4 μM HPRP-A1 for 12 h. kla co-administrated with HPRP-A1 exhibited low toxicity in NIH3T3 cells, but significantly decreased the viability of MCF-7 and A549 cells. The cell viabilities of MCF-7 and A549 cell lineswere around 60% and 10%, respectively, after incubation with 4 μM HPRP-A1 and 125 μM kla, while, the data on NIH 3T3 cell line was almost 100%. The cell viability of MCF-7 cell line decreased significantly along with the increasing concentration of kla peptide, in contrast, it declined slowly on A540 cell line, implying that the MCF-7 cell line was more sensitive to the peptide combination. Although a high concentration of kla alone exerted cytotoxic effects against cancer cells, there was stronger inhibition induced by kla with HPRP-A1 compared with kla alone as shown in (Fig 1).

### Cellular uptake of peptides

**Fig 2:**
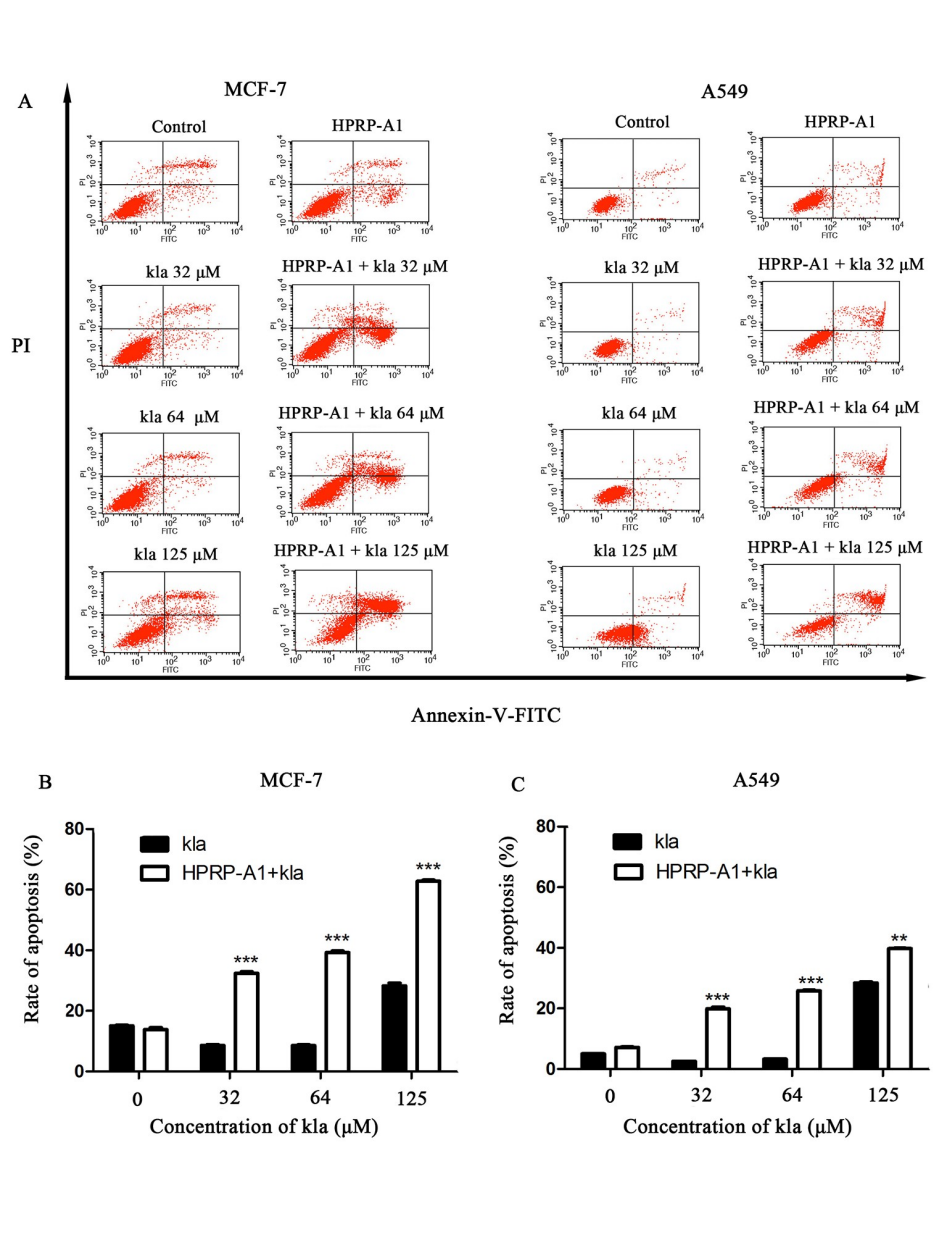
Cellular uptake of co-administration peptides by MCF-7 cells. The blue colour was nuclei stained by Hoechst33258, green colour was FITC-labelled kla. MCF-7 cells were treated with various concentrations of FITC-kla with or without 4 μM HPRP-A1 for 1 h. Fluorescence was measured by confocal microscopy.

In order to evaluate if HPRP-A1 could assistant kla transfer into cytoplasm and trace the intracellular distribution, the cellular uptake and colocalization of peptides were detected with FTIC-labelled kla peptide using confocal microscope. No green fluorescence was detected in the cell area in kla group, indicated that no FITC-kla were uptaken even in the concentration of 64 μM. However, after co-administration with 4 μM HPRP-A1, the bright green fluorescence was observedeven in the concentration of 16 μM group as shown in (Fig 2), indicated that FITC-kla effectively entered into MCF-7 cells by co-administrating with HPRP-A1.

**Fig 3:**
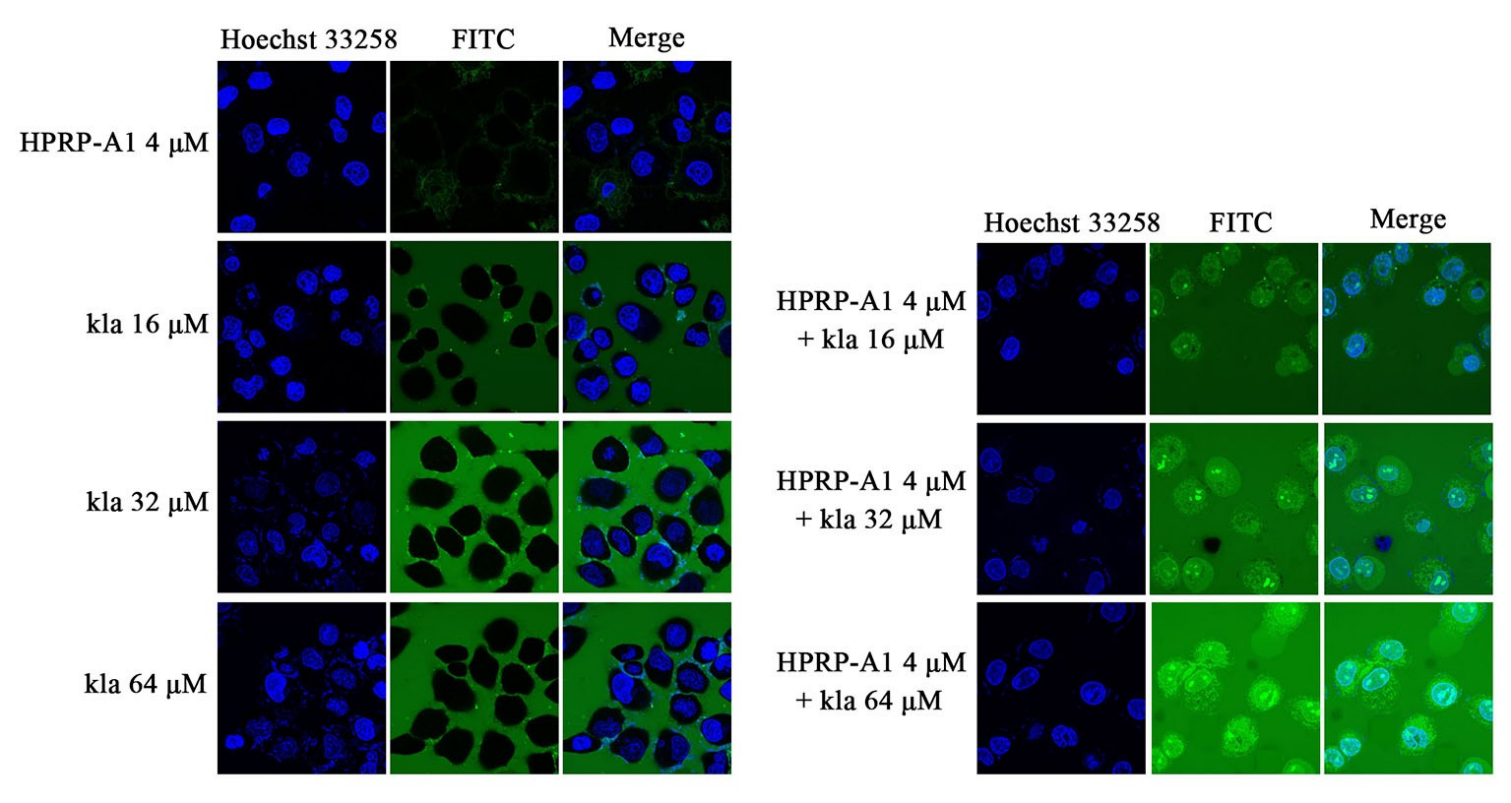
Co-localization of peptides in MCF-7 cells. Various concentrations of FITC-kla with or without 4 μM HPRP-A1 were applied to MCF-7 cells for 1 h. The blue colour was nuclei stained by Hoechst 33258, green colour was FITC-labelled kla and red colour was mitochondria stained by Mito-Tracker^®^ Red, respectively. Images were obtained by confocal microscopy.

Single peptide kla showed negligible binding to MCF-7 cells, whereas more kla peptides were absorbed when fused to HPRP-A1 as shown in (Fig 2). Internalization of kla has been shown to play a critical role in its cytotoxicity. To explore the mechanism underlying the cytotoxicity induced by kla with HPRP-A1, we investigated whether this peptide was internalized in mitochondria of MCF-7 cells using fluorescence confocal microscopy as shown in (Fig 3). Following incubation with MCF-7 cells, confocal microscopic images revealed that FITC-labelled kla was effectively taken up into MCF-7 cells and localized to mitochondria in a highly specific manner. The merged images showed near complete overlapping of FITC-labelled kla and Mito-Tracker signals, indicating that FITC-labelled kla was mainly localized at the mitochondrial membrane.

### Apoptosis analysis by flow cytometry

**Fig 4:**
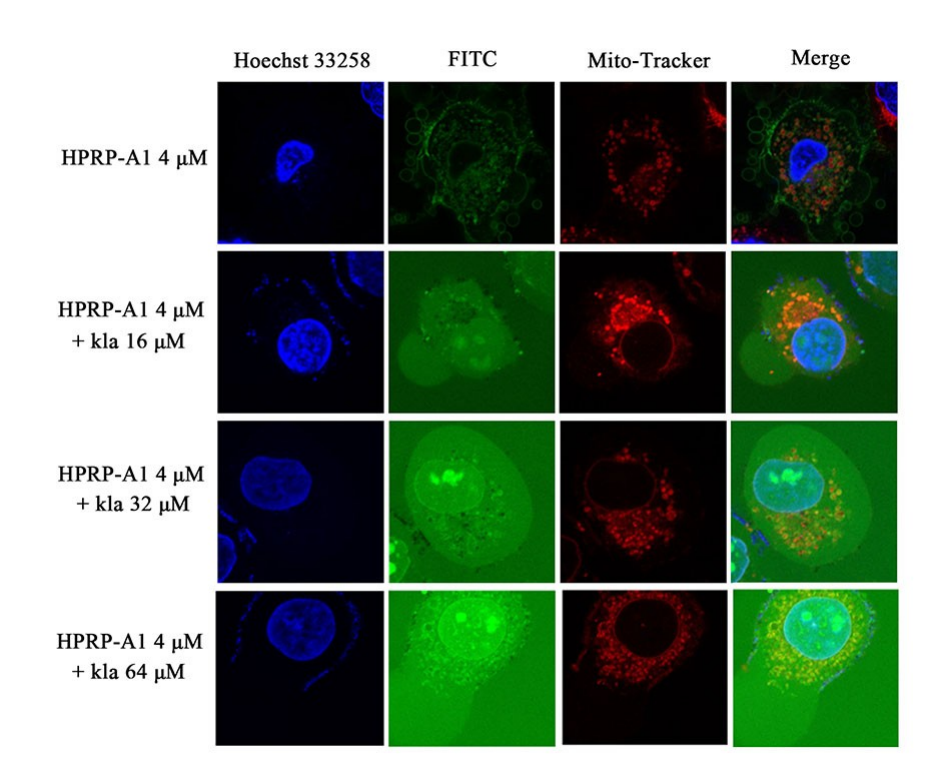
Apoptosis induced by kla with or without HPRP-A1. (A) MCF-7 and A549 cells were treated with various concentrations of kla with or without 4 μM HPRP-A1 for 12 h, and then the rates of early and late apoptosis of (B) MCF-7 and (C) A549 cells were measured using a FITC-Annexin V/PI kit and flow cytometry (***P<0.001; **P<0.01).

The peptide kla was an apoptosis inducing peptide, it could induce apoptosis after entering in the eukaryocyte[11, 21]. Meanwhile, early apoptosis inducing was one of the anticancer mechanisms of HPRP-A1, so, after co-administrating of the two peptides, the apoptosis rate of the cancer cells would be a critical parameter for evaluating the anticancer efficient *in vitro*. Theresult as shown in (Fig 4) indicated that the apoptosis rate of MCF-7 and A549 cells treated with co-administration peptides was significantly higher than that of kla and HPRP-A1 single peptide, *P<0*.*05* and *P<0*.*01* respectively.

### Mitochondrial depolarization

**Fig 5:**
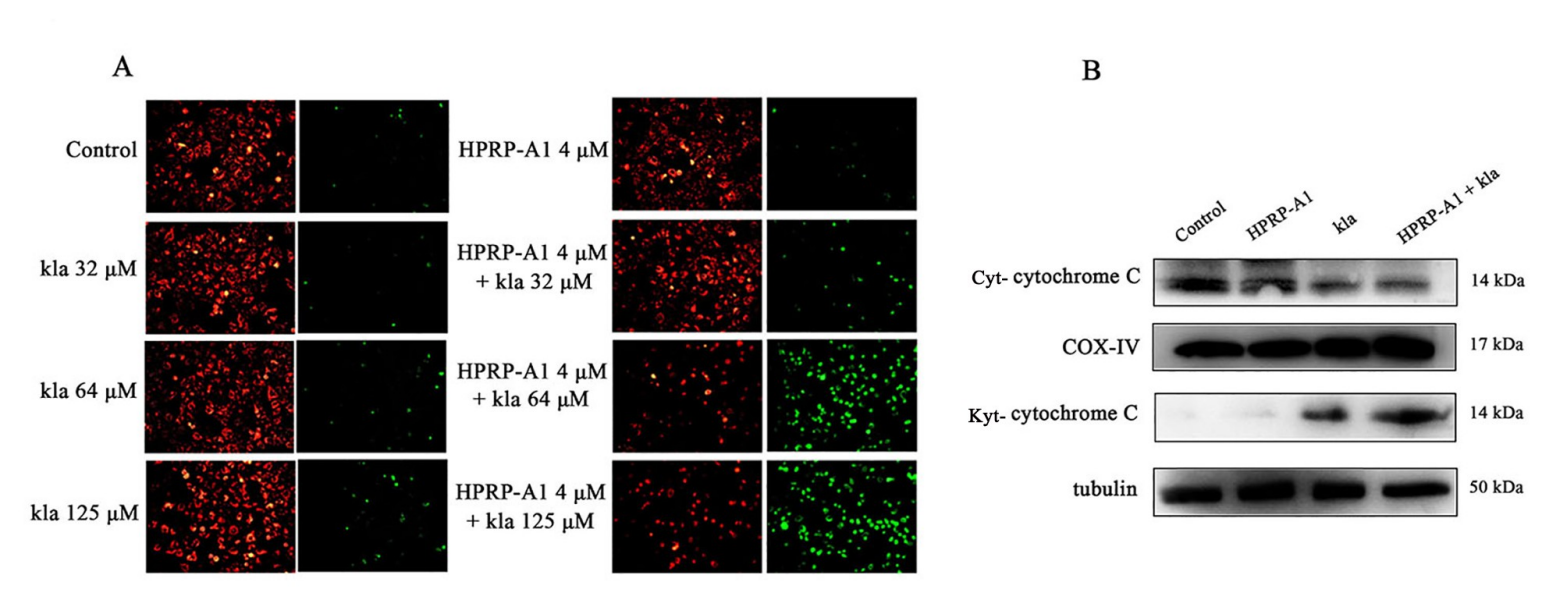
Mitochondrial depolarization and protein level of cytochrome C. (A) MCF-7 cells were treated with various concentrations kla with or without 4 μM HPRP-A1.MitoCapture has aggregated in the mitochondria of healthy cells shown as red. In apoptotic cells, MitoCapture cannot accumulate in mitochondria, which remains as monomers in the cytoplasm and fluoresces green. (B) Protein level of cytochrome C detected by western blotting in MCF-7 cells after treatment with kla with or without HPRP-A1 for 6 h. COX IV served as a mitochondrial reference control, and tubulin served as a cytoplasmic reference control.

To investigate the apoptosis mechanism of co-administration peptides, the mitochondrial membrane potential was measured by the MitoCapture regent which detecting the mitochondrial membrane potential by changing the colour of fluorescence. As shown in (Fig 5A), red fluorescence represents healthy cells and green colour represents mitochondrial depolarization cells, the result indicated us single kla and HPRP-A1 peptide did not lead to mitochondrial depolarization, however, after co-administrating with 4 μM HPRP-A1, 64 and 125 μM kla could result in remarkable mitochondrial depolarization.

An increase of cytochrome C release is also an indicator of mitochondrial membrane damage[22–24]. The result showed in (Fig 5B) revealed that the cytochrome C expression in mitochondrial was decreased in kla co-administration with HPRP-A1 group compared with the single peptide group, however, that in cytoplasmic (Cyt-cytochrome C)was increased in combination group. The result indicated that the cytochrome C in mitochondrial (Cyt-cytochrome C) was released from mitochondrial to cytoplasmic. In other words, kla induced cytochrome C releasing by co-administration with HPRP-A1, which would be a mechanism of apoptosis of the peptides.

### *In vivo* antitumor efficacy

**Fig 6:**
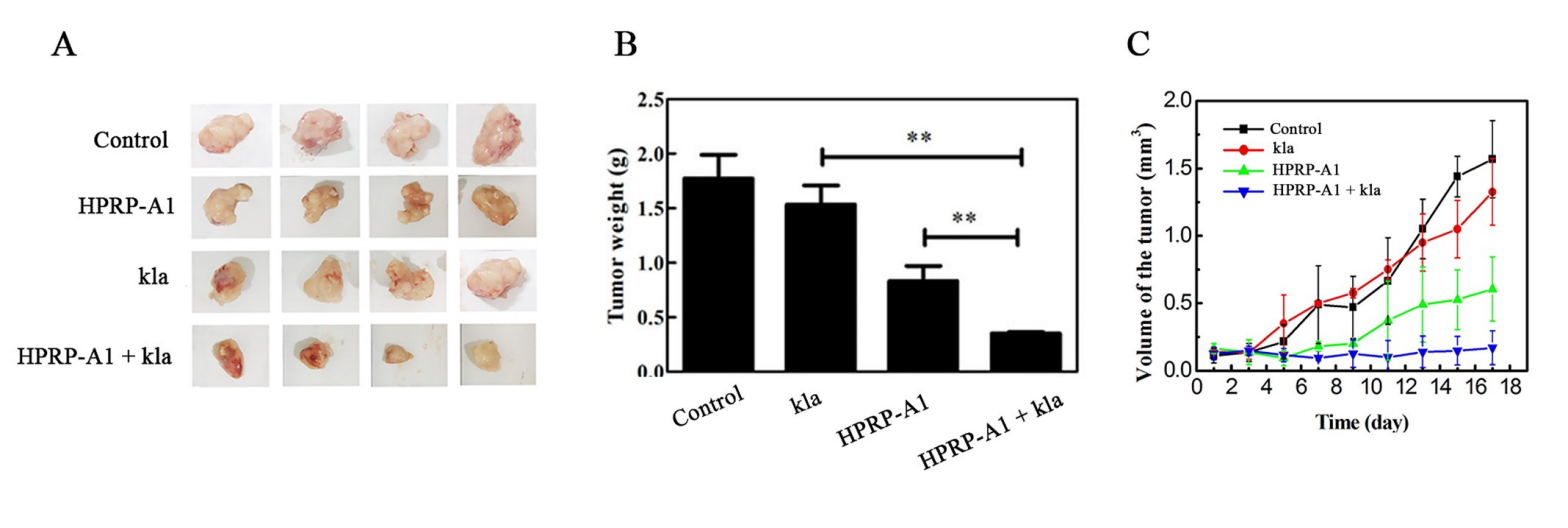
Antitumor activity of kla and HPRP-A1 *in vivo*. (A) Photographs of tumours. (B) Tumour weights. (C) Changes in tumour sizes.

To examine the in vivo antitumor effects of kla co-administrated with HPRP-A1, the peptide of kla with or without HPRP-A1weresystemically administered to MCF-7 nude xenograft mice. The peptides of HPRP-A1 and kla alone group and the co-administration group were directly injected into tumors once every 2 days. The control group was injected saline solution. The tumor volume were recorded every 2 days. The tumours were photographed (Fig 6A), the tumors volume in HPRP-A1 coadministration with kla group were dramatically smaller than that in HPRP-A1 and kla along group. The weights of tumors were showed in Fig 6B, the tumor weight in HPRP-A1 mixing with kla group was significantly lower than that in HPRP-A1 and kla alone group (P<0.01). The result of the volumes of tumor were shown in Fig 6C, the tumor volumes was up to 1.6 cm^3^ in control group, while, the volume was around 0.6 cm^3^and 0.2 cm^3^ in HPRP-A1 group and HPRP-A1 plus kla group.

**Fig 7:**
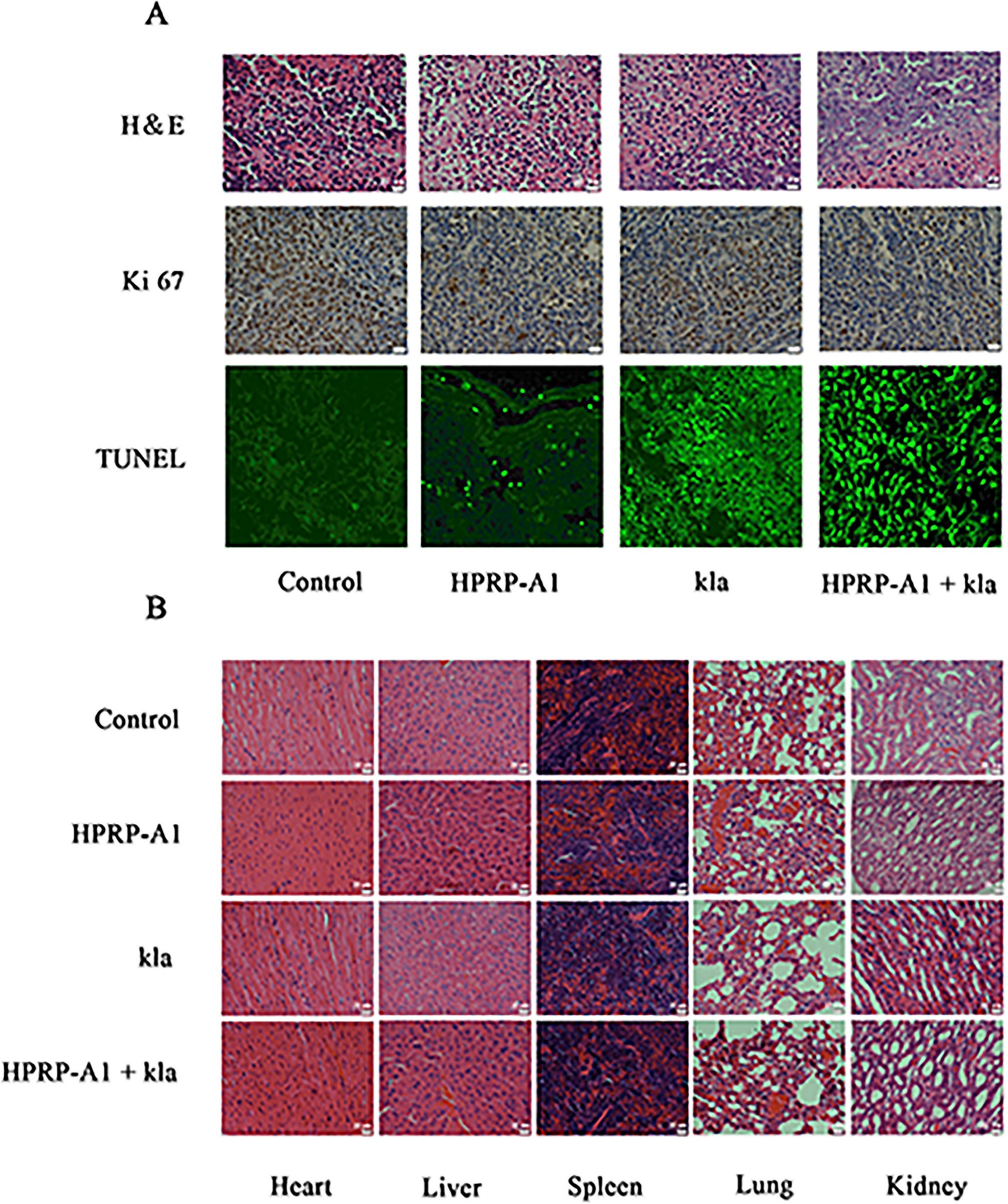
H&E, TUNEL and Ki 67 staining in tumour tissues and histological examination of major organs. (A) H&E, Ki67, and TUNEL staining of tumour tissues. (B) Histological examination by H&E staining of major organs (heart, liver, spleen, lungs, and kidneys) in the breast cancer mouse model.

The H&E assay, TUNEL assay and ki 67 expression of the tumor tissue were also investigated, the results were showed in Fig 7. As shown in Fig 7A, the quantity of the live cells in HPRP-A1 coadministration with kla treated group was less than that in other groups, indicating more cancer cells in tumor tissue were killed by the combination of peptides. The expression of ki 67 in HPRP-A1 group and HPRP-A1 plus kla group was lower than that in control and kla group. The result showed the combination peptides inhibited the proliferation of cancer cells. The TUNEL result suggesting a stronger apoptosis inducing ability of the combination peptides compared with the single peptide groups.

The H&E result of the primary organs (hear, liver, spleen, lung and kidney) in each group showed in Fig 7B were used to evaluate the potential toxicity of HPRP-A1 coadministration with kla. The results indicated that there were no obvious changes in these organs after treatment with single or combination peptides.

Haematoxylin-eosin staining (H&E), TUNEL and Ki67 expression in tumour tissues of all groupsweremeasured as well. The necrotic area in tumour tissue from mice treated with kla co-administrated with HPRP-A1 was much larger than that in tumour tissue from mice treated with kla alone. As shown in (Fig 7A), Ki67 expression in the mixed peptide group was significantly lower than that in control and single drug alone groups. TUNEL assays revealed many more apoptotic cells in tissues from mice treated with kla co-administrated with HPRP-A1 compared with those in tissues from mice treated with kla alone as shown in (Fig 7B). To examine whether HPRP-A1 enhanced adverse effects of kla, histological changes in normal tissues were assessed by H&E staining. In mice treated with kla co-administrated with HPRP-A1, there were no apparent histological changes in tissue sections of the heart, liver, spleen, lungs, and kidneys compared with the control group.

## Discussion

In recent years, anticancer peptides have made great progress. However, most anticancer peptides have limitations in their anticancer activities.kla, a polypeptide that specifically targets mitochondria and causes mitochondrial apoptosis, is poorly uptake by eukaryote cells,thus, it requires external assistance to enter cells[11, 25–27]. In the previous studies, HPRP-A1 exhibits promising anticancer activity through two mechanisms: cell membrane destruction and apoptosis induction[1, 28]. In this study, low concentration of HPRP-A1 assists kla to enter cancer cells without affecting the integrity ofcells, as a result, showing significantly synergistic anticancer activity. Compared with normal cells, cancer cells have more contents of anion components in their outer membrane, such as phosphatidylserine (3%-9% of total phospholipids), glycosylated mucin, sialic ganglioside, and heparin sulphate[8]. HPRP-A1 and kla can easily bind to cancer cytoplasmic membrane due to the electrostatic interactions. However, owing to the characteristic of inducibleα-helical structure of HPRP-A1[14], HPRP-A1 exhibits greater ability to further interact with phospholipids of membrane, resulting in the loose membrane structure, thus, helps kla to enter cells.

By comparing the viabilities of cancer and normal cells, coadministration of HPRP-A1 and kla showed dramatic killing effect on cancer cells and negligible toxicity in normal cells. For cationic anticancer peptide HPRP-A1, it can rely on its own net charge to recognize cancer cells and destroy the cell membrane leading to cell necrosis. At the same time, its hydrophobic and alpha-helix structure makes part of its polypeptides enter the mitochondrial enrichment of cancer cells and induce mitochondrial apoptosis. Previous studies on HPRP-A1 in our laboratory also showed that the higher the concentration, the stronger the toxicity[14, 15, 29]. Therefore, the concentration of the anticancer peptide HPRP-A1 co-administration was selected based on the safety of the drug, so that it can play a role in identifying cancer cells while changing the permeability of the membrane of cancer cells. Here, the concentration of 4 μM HPRP-A1 was selected. In addition to reducing drug toxicity, it can increase the uptake rate of anticancer peptide kla in cancer cells, so as to better play the role of the two peptides co-administration. In addition, kla is induced by targeting mitochondrial pathways of apoptosis[30]. All activities in the process of research results show that total dosing way anti-cancer effect is much higher than alone, and have kla concentration dependence, so we are based on the mechanism of the kla, made of mitochondrial apoptosis through experiments to verify our peptide drug delivery way adds to the feasibility of antitumor activity. The apoptosis rate induced by HPRP-A1 coadministration with kla peptide could reach 65% and 40% on MCF-7 and A549 cell lines, respectively. The results were consistent with the cell viability results showed in Fig 1, which verified the MCF-7 cell line was more sensitive to the peptide combination compared with the A549 cell line. The laser confocal co-localization experiment, mitochondrial membrane potential experiment and the detection of cytochrome C release by western blot indicated that the polypeptide kla played a role in inducing mitochondrial apoptosis in the co-administration mode. Subsequent *in vivo* animal and immunohistochemical analyses confirmed the tumour treatment effect of peptide coadministration together with low toxicity.

## Conclusion

In this study cationic membrane active anticancer peptide HPRP-A1 and anticancer peptide kla were administered together to increase the anticancer activity of polypeptide kla. Among them, cationic membrane active peptide HPRP-A1 plays a role in recognizing cancer cells and increasing their membrane permeability, so that kla peptides could enter into cytoplasm, induce mitochondrial depolarization, result in cytochrome C and promote apoptosis. More importantly, the co-administration peptides effectively inhibited the tumour growth in vivo, and have low toxicity to normal organs to the xenograft mice. HPRP-A1 and kla effectively improved the therapeutic activity of kla alone against cancer cells, which provides a research basis for development of effective treatment methods for cancer.

## Supporting information

S1 Fig High performance liquid phase diagram and flight mass spectrometry of polypeptides (A) kla and (B) HPRP-A1.
(TIF)

S2 Fig Circular dichroism spectra of peptides. Circular dichroism spectra of peptides (A) in benign medium at 25 *°*C and (B) in the presence of 50% TFE at 25°C.
(TIF)

S1 Table Hemolytic (MHC) activities of peptides kla, HPRP-A1 and HPRP-A1 with kla. (DOCX)
