# Supplementary figures and images for: Coadministration of kla peptide with HPRP-A1 to enhance anticancer activity
Source: PLoS One. 2019 Nov 8;14(11):e0223738. doi: 10.1371/journal.pone.0223738 (PMC6839859; doi:10.1371/journal.pone.0223738)

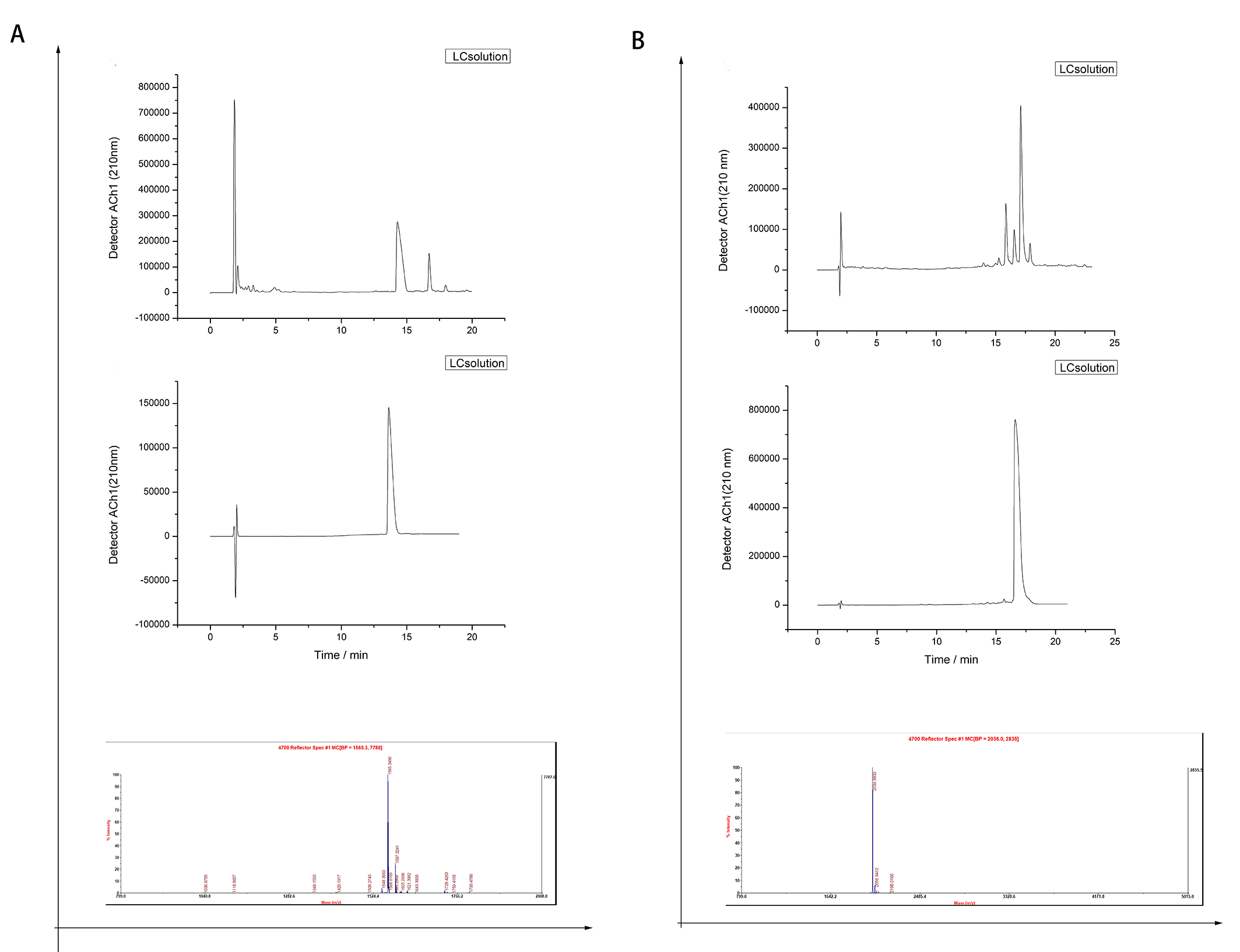

Supplement: S1 Fig — High performance liquid phase diagram and flight mass spectrometry of polypeptides (A) kla and (B) HPRP-A1. (TIF) [file pone.0223738.s001.tif]

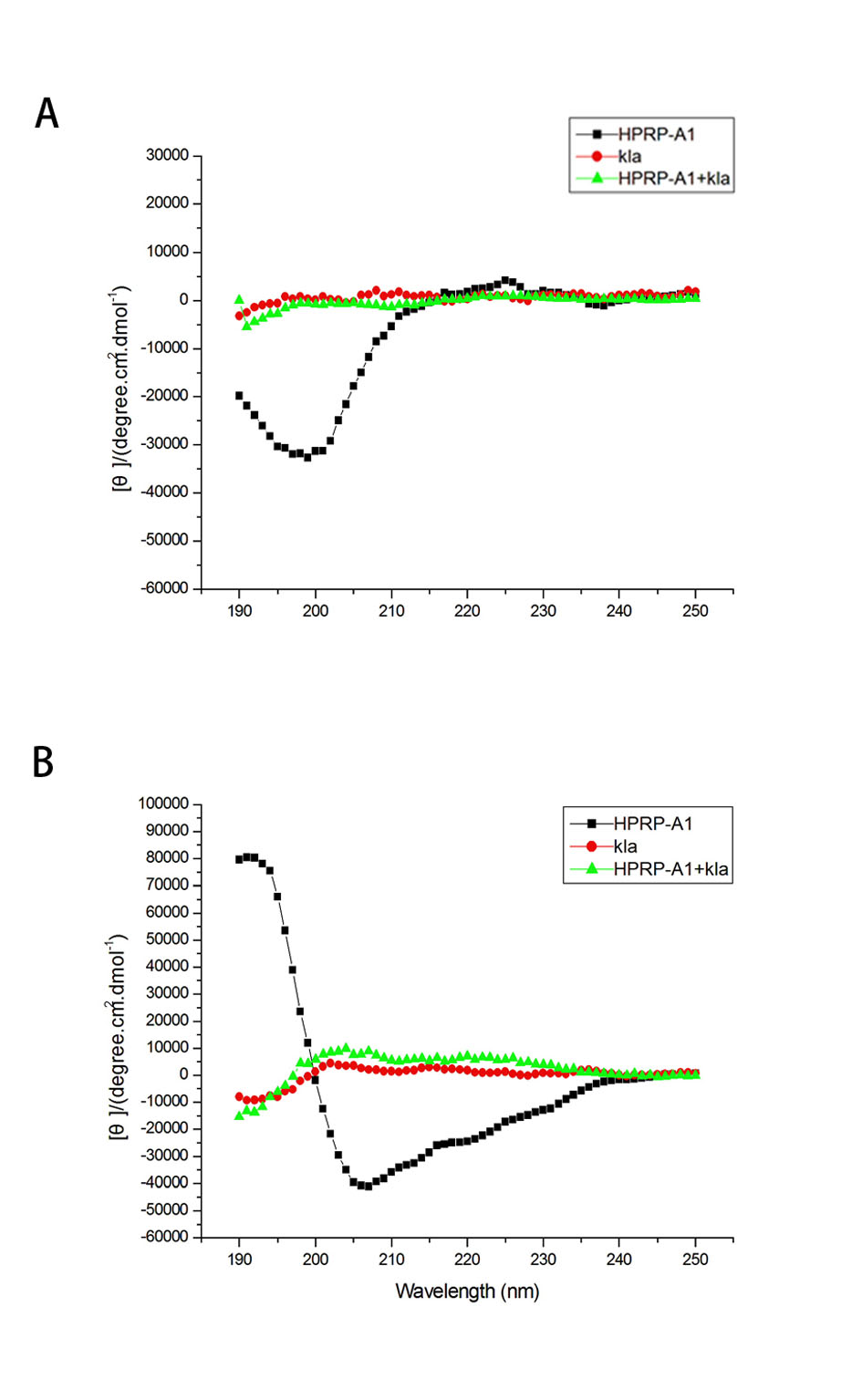

Supplement: S2 Fig — Circular dichroism spectra of peptides. Circular dichroism spectra of peptides (A) in benign medium at 25 °C and (B) in the presence of 50% TFE at 25°C. (TIF) [file pone.0223738.s002.tif]
