# Supplementary material for: Coadministration of kla peptide with HPRP-A1 to enhance anticancer activity
Source: PLoS One. 2019 Nov 8;14(11):e0223738. doi: 10.1371/journal.pone.0223738 (PMC6839859; doi:10.1371/journal.pone.0223738)
Supplement: S1 Table — (DOCX) [file pone.0223738.s003.docx]

S1 Table Hemolytic (MHC) activities of peptides kla, HPRP-A1 and HPRP-A1 with kla

| Peptide | MHC (μM) |
| --- | --- |
| kla | >250 |
| HPRP-A1 | >250 |
| HPRP-A1+kla | 64 |
